# Urinary cytokine profiles in unilateral congenital hydronephrosis

**DOI:** 10.1007/s00467-012-2230-9

**Published:** 2012-06-29

**Authors:** Katarzyna Taranta-Janusz, Anna Wasilewska, Wojciech Dębek, Marlena Waszkiewicz-Stojda

**Affiliations:** 1 Department of Pediatrics and Nephrology, Medical University of Białystok, 15-274 Białystok, Waszyngtona 17, Poland; 2 Pediatric Surgery Department, Medical University of Białystok, Białystok, Poland

**Keywords:** Monocyte chemotactic protein-1, Osteopontin, Regulated upon activation normal T-cell expressed and secreted chemokine, Ureteropelvic junction obstruction

## Abstract

**Background:**

We aimed to evaluate possible clinical application of urinary monocyte chemotactic protein-1 (MCP-1), osteopontin (OPN), and regulated upon activation, normal T-cell expressed and secreted (RANTES) chemokine as useful noninvasive markers in children with congenital hydronephrosis (HN) caused by ureteropelvic junction obstruction (UPJO).

**Methods:**

The study cohort consisted of surgical cases (study group 1), comprising 15 children with severe HN who required surgery (median age 1.03 years), conservative cases (study group 2), comprising 21 patients with mild, nonobstructive HN, and control group, comprising 19 healthy children. All children had normal renal function. Urinary (u) concentrations of MCP-1, OPN, and RANTES were measured using immunoenzymatic enzyme-linked immunosorbent assay (ELISA) commercial kits and were expressed in nanograms per milligram creatinine. Increased levels of MCP-1 and OPN were found in children with HN in comparison with study group 2 and controls (*p* < 0.05). UMCP-1/Cr correlated with half-time (T_1/2_) of the elimination phase of tracer excretion of technetium-99m-mercaptoacetyltriglycine (^99m^Tc-MAG-3) (*p* < 0.05).

**Results:**

Receiver operator characteristic (ROC) analyses revealed good diagnostic profile for uMCP-1 only in identifying children (<40 %) with abnormal differential renal function (DRF) [area under the curve (AUC) 0.862], and in detecting kidney injury in all examined children (AUC 0.704).

**Conclusions:**

Additional studies with larger number of patients are required to confirm a potential application of uMCP-1 as a promising parameter for early identification of kidney obstruction.

## Introduction

Hydronephrosis (HN) is defined as distension to varying degrees of pelvis and calyces accompanied by progressive atrophy of renal parenchyma due to obstruction in urinary flow [1]. Neonatal HN has been detected with increasing frequency with the widespread use of prenatal ultrasonography (US), but consensus about its postnatal management has not yet been reached, especially in relation to surgical intervention. Many neonates with renal dilation, usually detected prenatally, have no obvious clinical problems and seem to grow and thrive normally. Whereas we know that some children might have demonstrable deterioration of renal function over time, we clearly do not know who they may be.

Despite decades of investigation, there remain a number of fundamental questions regarding the evaluation and management of the fetus, infant, and child with obstructive nephropathy (ON). There is a need for improved methods to evaluate patients with congenital obstructive nephropathy, to time intervention, and to track progression and response to therapy. Plasma creatinine (Cr) concentration and nuclide scans are not satisfactory for these purposes, and new biomarkers are urgently needed. Whereas the ultimate goal is to find new therapies to minimize progression of renal injury, the interests of clinicians have focused on the potential role of plasma or urine markers, such as monocyte chemotactic protein-1 (MCP-1), osteopontin (OPN), and regulated upon activation normal T-cell expressed and secreted (RANTES) chemokine.

MCP-1, a powerful and specific chemotactic and activating factor for monocytes/macrophages and T lymphocytes [2], has been proposed as a possible mediator of tubulointerstitial lesions that follow endocytosis of filtered proteins. It is expressed by tubular epithelial cells, infiltrating monocytes, lymphocytes, and peritubular capillary endothelial cells in different renal diseases. Up-regulation of the gene for MCP-1 depends on activity of the transcription factor nuclear factor kappa B (NF-ĸB) [3], which is present in the inactive form in the cytoplasm of most cells and is activated upon proteolytic degradation of the inhibiting subunit I ĸB [4]. Among NF-ĸB-dependent genes, the chemokine RANTES, which has potent chemotactic activity for macrophages, granulocytes, and T lymphocytes, appears of particular interest in this context. RANTES is expressed by a variety of cell types, including lymphocytes, fibroblasts, mesangial cells, and renal tubular epithelial cells [5].

Osteopontin (OPN) is glycosylated phosphoprotein that mediates cell adhesion and migration and is produced by bone, macrophages, and endothelial and epithelial cells [6]. The many regulatory functions of OPN include bone remodeling, tumor invasion, wound repair, and promotion of cell survival. The role of OPN in modulating renal injury is unclear, with evidence for both inflammatory and anti-inflammatory actions [7]. Stimulation of renal OPN production by ureteropelvic junction obstruction (UPJO) may result at least in part from increased intrarenal angiotensin generated by mechanical stretch of dilated tubules.

We evaluated urinary MCP-1, OPN, and RANTES in children affected by severe congenital HN caused by UPJO. Additionally, we examined possible clinical application of urinary MCP-1, OPN, and RANTES as non invasive diagnostic and predictive biomarkers in UPJO.

## Patients and methods

This was a case-control prospective study performed in children with HN caused by UPJO who were diagnosed at the Department of Pediatrics and Nephrology and treated at the Pediatric Surgery Department of Medical University of Białystok, Poland. The study was performed from November 2010 to December 2011. Healthy children were chosen from those referred to our department because of miction disturbances and who were not on medications. Children were categorized into two study groups (1 and 2). The first group comprised 15 surgical cases (nine boys, six girls), median age 1.03 (0.08–14) years with severe HN due to a unilateral, critical degree of ureteral stenosis and who underwent pyeloplasty by the Anderson–Hynes method. The criterion adopted for inclusion in study group 1 was differential renal function (DRF) <40 % and/or anterior–posterior diameter (APD) of the renal pelvis on US scan >40 mm and half-time (T_1/2_) of the elimination phase of technetium-99m-mercaptoacetyltriglycine (^99m^Tc-MAG-3) diuretic renogram > 20 min. In this group, urine samples were collected three times: (1) first morning-voided urine samples obtained preoperatively, (2) urine samples collected directly from the obstructed pelvis, and (3) first morning–voided urine samples collected 3 months after surgery. The study group 2 consisted of 21 conservative cases (13 boys, eight girls; median: 8.0 years) with mild, nonobstructive HN who did not require surgery. Those children had suspected but not confirmed UPJO. The control group contained 19 patients (11 boys, eight girls; median 3.0 years) who were healthy without urinary dilation on US. Health status was determined through patients’ medical history and routine laboratory examinations to rule out the presence of acute or chronic disease.

Interpreted clinical features were age, gender, laterality, and renal function [serum creatinine, urea, and glomerular filtration rate (GFR) estimated by the Schwartz formula]. Patients were followed with clinical examinations, urinalysis, US, and renoscintigraphy. The degree of HN was graded according to the Society for Fetal Urology (SFU) classification [8]. Vesicoureteral reflux (VR) was ruled out in all patients by voiding cystourethrography (VCUG). Diagnosis of nonobstructive HN was made when the differential renal function was >40 % and/or stationary HN was found on US.

Criteria for inclusion in the study group were: (1) age 1 month–18 years, (2) signed informed consent obtained from the parents of the children, (3) unilateral pelvicaliceal system (PCS) dilatation, (4) a^ 99m^Tc–MAG-3 diuretic renogram (furosemide washout test) suggesting unilateral UPJO (study group 1), and (5) DRF if available. DRF of the affected dilated kidney of ≥ 45 % was considered normal and < 45 % abnormal. T_1/2_ tracer excretion of^ 99m^Tc–MAG-3 was used as an addition criterion for surgical intervention. T_1/2_ <10 min was considered as a normal response; T_1/2_ >20 min suggested obstruction. Values between 15 and 20 min were considered indeterminate [9]. Exclusion criteria were associated anomalies, including vesicoureteral reflux; ureterovesical junction and posterior urethral valve obstruction; bilateral HN; previous operation on the urinary system and other deformities of the external genital organs; deformities in the lower part of the ureter, bladder, and urethra, urinary stones; and neurogenic bladder dysfunction.

Urine was collected between 7 and 8 a.m., MCP-1, OPN, and RANTES measurements were performed in freshly voided midstream samples frozen at –80 °C. Patients with urinary tract infection were excluded. MCP-1 was measured using enzyme-linked immunosorbent assay (ELISA) with the commercially available kit (Bender MedSystems GmbH^®^, Vienna, Austria), according to manufacturer instructions. Urinary MCP-1 (uMCP-1) levels were expressed in picograms per milliliter. The detection limit was 2.31 pg/ml. The mean intra- and interassay coefficients of variation were 4.7 % and 8.7 %, respectively. OPN was measured using ELISA commercially available kit (R&D Quantikine, Abingdon, UK). All specimens were diluted to obtained concentration for optimal density according to instructions. Enzymatic reaction was quantified in an automatic microplate photometer. Urinary OPN (uOPN) level was expressed in picograms per milliliter. The detection limit was 0.011 pg/ml. Achieved results of plasma OPN (pOPN) were converted to nanograms per milliliter (ng/ml). Mean intra- and interassay coefficients of variation were 6.0 % and 5.9 %, respectively. RANTES was quantified by ELISA using a commercially available kit (Ray Bio® Human RANTES ELISA Kit). The lowest detectable concentration was 3 pg/ml. Mean intra- and interassay coefficients of variation were 10.0 % and 12.0 %, respectively. Urinary creatinine (Cr) concentration was used to normalize all parameter measurements to account for the influence of urinary dilution or concentration. The study was approved by the ethics committee of the Medical University of Białystok in accordance with the Declaration of Helsinki.

### Statistics

Data analysis was performed using computer program Statistica ver. 9.0 (StatSoft Inc., Tulsa, OK, USA). Nonparametric statistics were chosen, as the patient population was relatively small. Statistical analysis was performed using the Mann–Whitney* U* test. Differences between treatments were analyzed by Friedman’s analysis of variance (ANOVA) for repeated measures. Receiver operating characteristic (ROC) curve was used to determine the cutoff values of uMCP-1, uOPN, and uRANTES that gave the best sensitivity and specificity. Correlations between variables were evaluated by the Pearson or Spearman test, as appropriate.

## Results

The demographic and clinical data for each group are summarized in Table 1. Boys were more frequently affected with HN than were girls, which is confirmed in the literature [10]. The left kidney was more commonly involved both in children with severe HN and mild, nonobstructive HN (10 and 11, respectively). DRF in surgical cases was significantly lower than in conservative cases (*p* < 0.01), and the APD of the renal pelvis was found to be significantly higher in study 1 group than in study 2 children (*p* <0.05). Renoscintigraphy was not performed in healthy children, and DRF was not assessed. T_1/2_ tracer excretion of ^ 99m^Tc–MAG-3 >20 min was observed in all study group 1 patients and in 12/21 (57.1 %) conservative patients.

**Table 1:** Summary of clinical parameters in all patients

Clinical parameters	Study group 1	Study group 2	Controls
	Median (range)		
No. (male/female)	15 (9/6)	21 (13/8)	19 (11/8)
Age at diagnosis (years)	0.25 (0.08–8.0)	5.0 (0.08–17)	–
Age at examination (years)	1.03 (0.08–14)	8.0 (0.75–17)	3.0 (0.33–16)
Clinical diagnosis (SFU grading)			
Grade 1	–	4	–
Grade 2	–	12	
Grade 3	6	5	
Grade 4	9	–	
Laterality (left/right)	10/5	11/10	–
Differential renal function (%)	45 (18.1–49)	45.8 (42–50)	–
Anterior-posterior diameter of affected renal pelvis (mm)	38.5	31.0	
	(16–70)	(21–41)	
Estimated glomerular filtration rate (ml/min/1.73 m^2^)	102.78	134.79	110.15
	(65.036–130.25)	(91.77–203.9)	(83.73–175.53)
T_1/2_ tracer excretion of ^ 99m^Tc–MAG-3 (min)	35.5	15.5	–
	(22.0–84.0)	(10.0–25.0)	

Values given in parenthesis are the ranges unless stated otherwise

*SFU* Simon Fraser University,* T*_*1/2*_ half-time, ^* 99m*^ *Tc–MAG-3* technetium-99m-mercaptoacetyltriglycine

UMCP-1/Cr levels from voided urine before and after surgery and concentrations obtained from the affected pelvis were significantly greater than in study 2 and control groups (*p* < 0.05). The difference between uMCP-1/Cr level from voided urine and affected pelvis was not statistically significant (*p* > 0.05). Three months after surgery, in comparison with exam B, uMCP-1/Cr level did not decrease (*p* > 0.05), and the difference in comparison with children from study 2 and control groups was significant (*p* < 0.05, *p* < 0.01, respectively) (Table 2) .

**Table 2:** Urinary concentration of monocyte chemotactic protein-1 (MCP-1), osteopontin (OPN), and regulated upon activation, normal T-cell expressed and secreted (RANTES) chemokine in patients with ureteropelvic junction obstruction (UPJO) from study groups 1 and 2 and healthy controls

Examination group^a^	uMCP-1	uOPN	uRANTES
	pg/ml pg/mg Cr	ng/ml ng/mg Cr	pg/ml pg/mg Cr
Exam A (voided urine before pyeloplasty)	62.14 (0.4–456.91)^A, a^	37.38 (2.77–38.57)^A, b^	7.45 (4.78–51.84)
	76.77 (0.45–1275.44)^A, a^	52.29 (5.5–413.4)	21.29 (3.27–113.48)
Exam B (urine samples from affected pelvis during surgery)	35.71 (1.72–197.79)^a^	36.62 (6.01–38.3)^a^	13.9 (6.8–84.33)^a^
	94.07 (2.57–438.63)^B,b^	73.1 (12.81–314.85)^B, b^	45.42 (13.45–233.65)^B, b^
Exam C (voided urine 3 months after surgery)	28.92 (2.95–125.04)^a^	35.16 (17.49–37.81)^b^	10.17 (5.57–35.61)
	56.1 (10.0–276.79)^A, b^	80.52 (21.97–279.79)^B, b^	31.11 (10.37–136.02)^A, b^
Study group 2 (children with suspected but not confirmed UPJO)	12.86 (2.21–79.09)	34.66 (1.59–37.8)^a^	14.43 (0.6–32.1)
	14.04 (1.73–498.69)	19.37 (0.86–57.51)	35.33 (1.17–184.9)
Control (healthy children)	10.11 (1.6–46.38)	33.92 (3.35–35.55)^A^	10.89 (5.97–21.17)
	9.17 (2.18–155.63)	39.32 (4.29–113.83)	13.2 (5.52–46.65)

Values are median (range)

*uMCP-1* urinary monocyte chemoattractant protein-1,* uOPN* urinary osteopontin,* uRANTES* urinary regulated on activation normal T-cell expressed and secreted chemokines,* Cr* creatinine,* UPJO* ureteropelvic junction obstruction

^A^ *p *<0.05 to study group 2, ^a^ *p *<0.05 to controls, ^B^ *p *<0.01 to study group 2, ^b^ *p *<0.01 to controls

Median uOPN/Cr level was significantly higher in patients from the study group 1 compared with conservative cases (*p* <0.01). There was no difference between urine collected before surgery and urine obtained from affected pelvis (*p* > 0.05). The values of uOPN/Cr found in urine obtained 3 months after pyeloplasty were similar to those observed in exam A and B (*p* > 0.05). Values in exam C were significantly higher than those observed in study 2 and control groups (*p* < 0.01). URANTES/Cr was significantly higher in exam B than preoperative levels in exam A (*p* < 0.05). Three months after surgery, no significant differences in values were noted in comparison with exam B (*p* > 0.05). Postoperative uRANTES/Cr were lower from values in study group 2 patients (*p* < 0.05) but much higher than in controls (*p* < 0.01).

A negative correlation between uMCP-1/Cr, uOPN/Cr, uRANTES/Cr, and DRF was found in the study group 1 (*p* < 0.05). The correlation was even stronger between uMCP-1/Cr from affected pelvis and DRF (*r* = -0.69, *p* < 0.001). No correlation was found between estimated parameters and DRF in study group 2 (MCP-1/Cr:* r* = -0.002, OPN/Cr:* r* = -0.07, uRANTES/Cr:* r* = -0.13, *p* > 0.05). We found no significant correlation with serum Cr level, age of patients, and GFR. We also found that increase in T_1/2_ tracer excretion >20 min was associated with increase in MCP-1 (above median value in controls) [odds ratio (OR) 3.8; confidence interval (CI) 0.84–17.0] and RANTES (OR 2.42; CI 0.57–10.1) but not in OPN (OR 0.96; CI 0.18–4.92). However, only uMCP-1/Cr correlated with T_1/2_ tracer excretion (*r* = 0.63, *p* < 0.05) (Table 3) .

**Table 3:** Statistically significant correlations between estimated markers and differential renal function (DRF) in children from study group 1 (Spearman correlation analysis)

	Correlation (*r*)	*P* value
MCP-1/Cr	−0.73	0.0001
OPN/Cr	−0.29	0.04
RANTES/Cr	−0.41	0.004

**Fig. 1:**
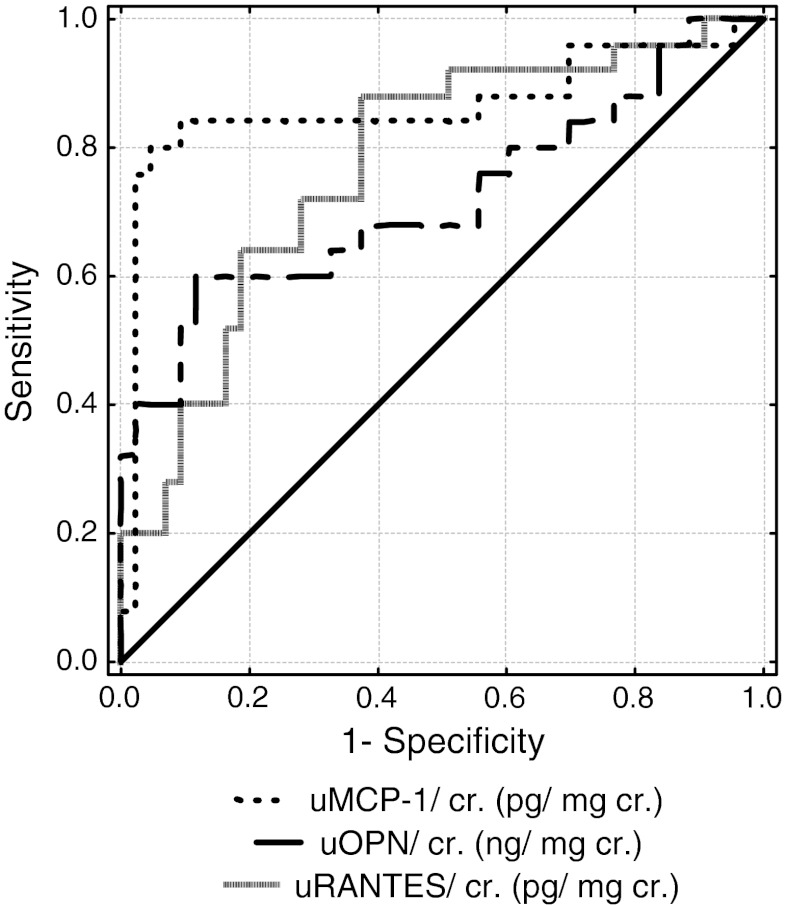
Receiver operating characteristic (ROC) curves for urinary monocyte chemotactic protein-1 (uMCP-1), osteopontin (uOPN), and regulated upon activation, normal T-cell expressed and secreted (uRANTES) chemokine levels in children with obstructive kidney [differential renal function (DRF) < 40 %)]among children with ureteropelvic junction obstruction (UPJO)

ROC analyses were performed to define the diagnostic profile of uMCP-1, uOPN, and uRANTES in identifying children with HN among all examined children. area under concentration time curve (AUC) for uMCP-1/Cr was 0.704 (CI 0.581–0.827), with a best cutoff value 0.45 pg/mg Cr (sensitivity 100 %, specificity 0 %). AUC for uOPN/Cr was 0.666 (CI 0.544–0.787), with a best cutoff value 5.502 ng/mg Cr (sensitivity 98.5 %, specificity 10.5 %). AUC for uRANTES/Cr was 0.693 (CI 0.58–0.807), with a best cutoff value 0.865 pg/mg Cr (sensitivity 100 %, specificity 0 %). In further analyses, we assessed AUC in children with obstructive kidney (DRF < 40 %) (Fig. 1). Only uMCP-1 ROC curves showed good diagnostic accuracy (AUC >0.8) in study groups 1 and 2 (AUC 0.862). ROC analyses for uMCP-1, uOPN, and uRANTES not corrected for uCr showed similar values. Neither MCP-1 nor OPN and RANTES revealed good diagnostic accuracy AUC in children with T_1/2_ >20 min.

## Discussion

Whereas there are no clear indications for timing of surgical intervention to prevent kidney injury, discovery of potential biomarkers of disease progression resulting from urinary tract obstruction could be useful in determining targets for therapeutic manipulation and monitoring. Recent papers showed that urine emerged as a potential and more suitable reservoir than blood for identifying biomarkers. In contrast to blood, preanalytical handling is simple, and urine is particularly stable. It is estimated that about 70 % of urinary proteins originate from the kidney and the urinary tract in healthy individuals, which might be even higher in individuals with kidney diseases [11–15]. That is why we are now looking for urinary-excreted markers of renal injury in UPJO children.

In this case–control prospective study, we compared the magnitude of alternation in uMCP-1, uOPN, and uRANTES concentration in urine at different time periods in children with confirmed HN with levels in children with mild, nonobstructive HN and healthy participants. Additionally, we evaluated the usefulness of uMCP-1, uOPN, and uRANTES as biomarkers of obstructive nephropathy. To our knowledge, this study represents the first published attempt to propose uMCP-1, uOPN, and uRANTES as a panel of biomarkers in congenital obstructive nephropathy.

The first part of our study was the classical biomarker approach. We demonstrated that urinary cytokines successfully discriminated not only between children with HN and controls but within the HN group. We found that both uMCP-1 and uOPN but not RANTES levels in voided urine in HN before surgical treatment were much higher than in study group 2 and control groups. UMCP-1 levels prior to pyeloplasty showed significant elevation in patients who developed obstructed kidney. Patients from the study group 1 revealed significant difference in uMCP-1 levels in comparison with study group 2 and control groups (*p* < 0.05). In study group 1, uOPN levels were about fourfold higher than in conservative cases and almost twofold greater than values in healthy participants.

The final diagnosis of UPJO was made using clinical algorithm with the following criteria: DRF <40 % and/or anterior–posterior diameter (APD) of the renal pelvis on US scan >40 mm and T_1/2_^99m^Tc-MAG-3 diuretic renogram > 20 min. We found positive correlation between uMCP-1/Cr with T_1/2_ tracer excretion (*p* < 0.05).

We also prospectively analyzed data of 15 patients enrolled in study group 1; however, the research period was quite short. UMCP-1 levels in patients who developed an obstructed kidney but had not yet undergone surgical correction of the obstruction were similar to values found in urine from the affected kidney and after pyeloplasty. The possible explanation of this fact may be not only the urine dilution effect from the contralateral kidney, but also the lower Cr excretion of the obstructed kidney with reduced glomerular function. There was no observed difference in uOPN/Cr values between urine collected before surgery compared with that obtained from the affected pelvis.

The third analyzed marker in this report, uRANTES, had also been studied extensively in different pathological conditions (i.e., kidney transplant patients, crescentic nephritis) [16, 17]. Interestingly, in our study, only RANTES concentration in pelvic urine from the affected kidney statistically exceeded values in voided urine before pyeloplasty (*p* < 0.05). Decreased urinary levels of postoperative uRANTES in children with UPJO might reflect a lower and more steady-state production of inflammatory cytokines with long-standing obstruction. It was also confirmed in the estimation of transforming growth factor (TGF)-beta 1 concentration in patients with upper urinary tract obstruction [18].

Three months after surgery, uMCP-1 concentration was statistically higher from values found in children with dilated, but not obstructed, kidney (study group 2) (*p* < 0.05). It is interesting that analysis of uMCP-1 in particular patients showed that the significant decrease was observed in patients with the highest levels. Similar observation was made by Grandaliano and colleagues [19], who analyzed both MCP-1 expression on renal biopsies and uMCP-1 concentrations in severe UPJO, revealing a fourfold higher uMCP-1 concentration in studied children than in healthy controls. Contradictory to our study, they reported markedly reduced uMCP-1 excretion after surgery, reaching normal levels 4 months after surgical correction of the obstruction. Increased uMCP-1 in diseased kidney was also reported by Lloyd et al. [16] using crescentic glomerulonephritis model. Those observations led to the proposal that uMCP-1 can be considered a noninvasive marker of kidney damage in children with UPJO. Their proposal is supported by data from our ROC analyses, which showed good diagnostic profile for uMCP-1 in identifying children with abnormal DRF (< 40 %) among all studied children (study groups 1 and 2; AUC 0.862) and quite a good profile in detecting kidney injury in all examined children (AUC 0.704). The results suggest that uMCP-1 may be useful in identifying the presence of obstructive nephropathy in HN children.

Our study also showed similar values of uOPN/Cr in urine obtained 3 months after pyeloplasty in comparison with exams A and B (*p* > 0.05). Values in exam C significantly exceeded values found in children with dilated but unobstructed kidney (study group 2) and in patients from the control group (*p* < 0.01). The possible reason for this situation is that endogenous renal OPN may serve a potentially protective role in tubules. In the report by Taha et al. [20], postoperative values of estimated endothelin-1 also remained greater than basal levels and intrapelvic urine concentrations in UPJO children. The authors suggest that it takes a long time for the kidney to acquire a steady phase after relief of obstruction, which seems to be confirmed in our study.

UMCP-1/Cr and uOPN/Cr bladder values and values from the affected pelvis were found to be so close together, and those data were significantly higher than in healthy controls. This is an unexpected finding and provides new insights into some of the current thinking on compensatory hypertrophy and tissue damage in an obstructed kidney. Gawlowska and Niedzielski [21] reported a preliminary study in which mean uRANTES concentration of 28 children operated due to UPJO was significantly higher than in controls. Our results also support this contention.

The ideal urinary biomarker is measurable in bladder urine. The need to obtain renal pelvic urine to improve the sensitivity of the biomarker necessitates an invasive procedure and is therefore less attractive. The biomarker must be highly sensitive, specific, and have a high predictive value. Peters et al. [22] show that urinary biomarkers of ureteral obstruction are different for infants and for children, and they suggest that reference ranges of urinary cytokine biomarkers of renal injury in UPJO need to be age adjusted. In our study, there was no correlation found between estimated parameters and patient age. Other authors show that protein markers for UPJO are predictive in infants but not older children. This may reflect a more varied diet in older children than in infants [23].

Most markers are affected by infection and is thus difficult to estimate, as all children in our study had no signs of acute infection. The well-known urinary biomarkers mentioned above may have the potential to be markers for diagnosing UPJO, and they may be useful in the postoperative follow-up of children with this abnormality. If the marker correlates with renal function, it will be a good marker of disease progression and may have the potential to predict which patients will require surgery. The lack of this correlation in our study might be explained by the fact that all children enrolled had normal renal function.

Our study has some limitations. One is low specificity of the biomarkers to obstructive nephropathy, as they can increase/decrease in other types of nephropathy [16]. Another is the 3-month follow-up, which is relatively short for urinary cytokine normalization and does not allow us to draw an unequivocal conclusion. Additional studies with follow-up US and renoscintigraphy data correlated to changes in urinary cytokine profiles might succeed in this mission.

In conclusion, we provide novel data documenting the increase in excretion of three examined factors. In this study, elevated levels of uMCP-1, uOPN, and uRANTES were detected. However, the study failed to identify the biomarker that might help predict which patients with UPJO would require surgical correction before deterioration of renal function. Nevertheless, uMCP-1 seemed to be promising noninvasive parameter for early detection of obstruction. However a panel of biomarkers, as a combination of markers increasing and decreasing with severity of obstruction, would be more helpful than a single marker.
